# Multiple Broadband Infrared Topological Photonic Crystal Valley States Based on Liquid Crystals

**DOI:** 10.3390/ma17215212

**Published:** 2024-10-25

**Authors:** Jinying Zhang, Bingnan Wang, Rui Wang, Jiacheng Wang, Xinye Wang, Yexiaotong Zhang

**Affiliations:** 1 Beijing Key Lab for Precision Optoelectronic Measurement Instrument and Technology, School of Optics and Photonics, Beijing Institute of Technology, Beijing 100081, China; 3120215361@bit.edu.cn (B.W.); 3120190641@bit.edu.cn (R.W.); 3120225346@bit.edu.cn (J.W.); 3120205353@bit.edu.cn (X.W.); 3120220661@bit.edu.cn (Y.Z.); 2 Yangtze Delta Region Academy of Beijing Institute of Technology, Jiaxing 314001, China

**Keywords:** liquid crystal, valley photonic crystal, dual band, valley states, terahertz

## Abstract

Spectral tunable technology has to meet the requirements of strong robustness and wide spectral range. We propose a method for the transmission and manipulation of infrared topological photonic crystal valley states based on tunable refractive index method that exhibits broad-spectrum and multi-band characteristics, along with a tunable emission angle. With this structure, different rotational directions of vortex light sources can independently excite the K valley and K′ valley within the frequency band ranging from 75.64 THz to 99.61 THz. At frequencies from 142.60 THz to 171.12 THz, it is possible to simultaneously excite both the K valley and K′ valley. The dual refractive index tunable design allows for the adjustment of the emission angle at a fixed frequency, enabling control over the independent excitation of either a single K valley or K′ valley, as well as their simultaneous excitation. This capability has significant implications for photonic computation and tunable filtering, offering enhanced operational flexibility and expanded functionality for future optical communications and integrated optical circuits.

## 1. Introduction

Spectral tunability technology [1] is urgently needed in the fields of photonic computing [2], fiber optic communications [3], and biomedical imaging applications [4]. Currently available spectrally tunable technologies include filter arrays [5,6], acousto-optic tunable technologies [7,8,9], liquid crystal tunable filters [10,11,12], photonic crystal metamaterials [13,14,15], and microelectromechanical systems (MEMS) wavelength tunable filters [16,17,18,19]. Guo et al. [20] presented a visible light wide-spectrum super Fabry–Pérot (F-P) filter capable of adjusting its color response by manipulating the polarization of the incident light, and this has a modulation range of approximately 300 nm. Rui et al. [21]. introduced a spectral tunability technology based on acousto-optic tunable filters (ATOF) which enable programmable spectral lines within a range from 0.45 to 0.95 μm. However, its development is significantly limited due to its strong dependence on both wavelength and incident angle for light diffraction. Shouta et al. [22] utilized the birefringence of liquid crystals by stacking liquid crystal layers with different birefringence wavelength dependencies, controlling their optical axes perpendicularly to each other for spectral control. By adjusting the voltage applied across the birefringent layers of the liquid crystals, the central wavelength and full width at half maximum (FWHM) of the spectrum could be controlled. This method enables the electronic control of both the spectral range and directivity, but imposes strict limitations on the incident light’s directionality. Additionally, the introduction of electrodes unavoidably affects the filtering performance, potentially limiting its practical applications.

Although the reported spectrally tunable technologies each feature wide spectral ranges and strong robustness, they required strict conditions for the incident light and output directions, and their wide spectral ranges still exhibit significant limitations. Since Vesna et al. [23] introduced the concept of path design for two-dimensional photonic crystal metamaterials to guide light paths, researchers have increasingly focused on this area. Two-dimensional topological photonic crystals are characterized by their robustness, strong directionality, and defect tolerance. Their unique edge states exhibit high compatibility with other systems, making them a prominent area of research in the field of spectral control in recent years [24,25,26,27,28].

By precisely designing the structure of photonic crystals, waves within specific bandgaps can propagate along predetermined directions, demonstrating strong robustness, directionality, defect tolerance, and anti-scattering capability. Wang et al. [29]. first proposed the tunable edge states of two-dimensional topological photonic crystals in triangular lattices and investigated their valley states. However, the sharp corners inherent to triangular lattices pose fabrication challenges. Once designed, the structure is confined to a single bandgap and fixed propagation path, with a very narrow wavelength tunability range, severely limiting its application scenarios. Reports on further expanding its spectral tunability are scarce, and many mechanisms require in-depth research.

This paper proposes a more easily fabricated triangular lattice topological photonic crystal structure [30], introducing a dual refractive index tunable spectral control technology. Verification of valley topological photonic crystal excitation at three distinct wave-lengths demonstrates that single liquid crystal refractive index control can broaden the excitation wavelength range, achieving strong excitation of dual-band beams. By modifying the refractive index of another control unit for dual excitation, the emission angle of the excitation light source can be altered, freely modulating the direction of the light path and enabling switching between different mode-induced light paths. The separate control by two control units allows for complex photonic crystal logic operations.

## 2. Materials and Methods

### 2.1. Dual-Band Topological Photonic Crystal

**Figure 1:**
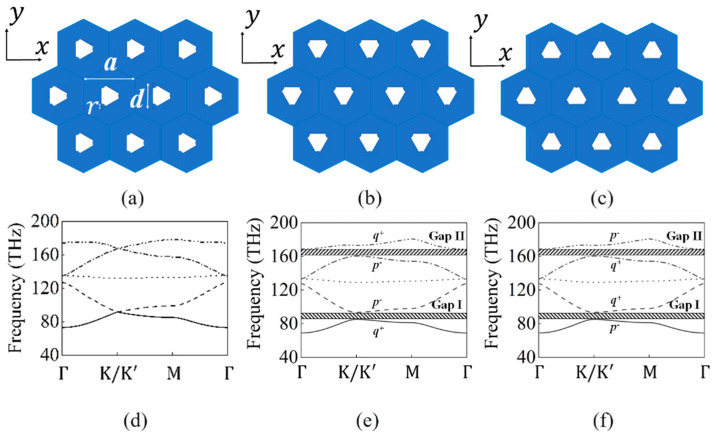
Schematic of two-dimensional photonic crystal structure with *C*_3*V*_ symmetry of arc-cut triangular units arranged in a triangle, and its band structure: (**a**) *θ* = 0° photonic crystal structure; (**b**) *θ* = 30° photonic crystal structure; (**c**) *θ* = −30° photonic crystal structure; (**d**) *θ* = 0° photonic crystal band structure; (**e**) *θ* = 30° photonic crystal band structure; (**f**) *θ* = −30° photonic crystal band structure.

In our previous research [30], we published a study on photonic crystals with a triangular arrangement and a *C*_3*V*_ symmetry structure that explored the supercell structure and band of the first and second edge states under zigzag- and armchair-type interfaces, thus verifying their robustness. The unit’s cell structure, as depicted in Figure 1a, consists of a scattering element (white region) formed by truncating sectors with a central angle of 60° from an equilateral triangle at each vertex. These sectors are centered at the vertices of the equilateral triangle and intersect with its boundaries. The scatterers are composed of metal, with perfect electric conductor (PEC) simulations used to design the interface between the scatterers and the background materials (blue region). The background materials employ a nematic liquid crystal mixture [31], NJU-LDn-4, whose refractive index ($n_{1}$) varies between 1.5 and 1.8 in the terahertz range, depending on the applied voltage. The unit cell structure is characterized by the following parameters: the lattice constant ($a=\sqrt{3}\;µm$), the side length of the equilateral triangle ($d=3\sqrt{3}a/10$), and the radius of the sector (r=0.1 d). Within the photonic crystal structure, the scatterers can rotate around the center of the unit cell. The angle of rotation, denoted by *θ*, is defined relative to the x-axis; positive values of *θ* correspond to counterclockwise rotation, while negative values indicate clockwise rotation. This photonic crystal structure exhibits *C*_3*V*_ symmetry and exhibits two degenerate points in its band structure, leading to the formation of two Dirac cone structures, as illustrated in Figure 1d. By rotating the scatterers by 30° counterclockwise and clockwise, respectively, as illustrated in Figure 1b,c, the spatial inversion symmetry is broken, changing the symmetry from *C*_3*V*_ to *C*_3_. This symmetry reduction lifts the degeneracy of the two points, resulting in the opening of two bandgaps within the band structure. The first bandgap is denoted as Gap I and the second as Gap II, with their band structures depicted in Figure 1e,f.

The Berry curvatures of the bands below Gap I and Gap II can be calculated using the k.p perturbation method [32,33] (where *k* represents the crystal momentum and p denotes the momentum operator), as follows:

(1) $$\Omega \left(\delta k\right)=m_{i}v_{Di}/[2{\left(\delta k^{2}+{m_{i}}^{2}+{v_{Di}}^{2}\right)}^{\frac{3}{2}}]$$

In the Equation (1), $v_{Di}$ represents the Dirac cone dispersion velocity at a rotation angle of θ=0°, where *θ* denotes the rotation angle, representing the orientational degree of freedom. δk is the momentum offset from the K point in the Brillouin zone and $\;m_{i}=(\omega_{q+}^{i}-\omega_{q-}^{i})/(2v_{Di}^{2})$ is the effective mass. Here, $\omega_{q+}^{i}$ corresponds to the band frequency for counterclockwise energy flow and $\omega_{q-}^{i}$ is the band frequency for clockwise energy flow. The valley Chern number is defined as the integral of the Berry curvature over the Brillouin zone, as shown in the following Equation (2) [34]:

(2) $$C_{K(K^{\prime })}=\frac{1}{2\pi }\int_{BZ}\Omega \left(\delta k\right)d^{2}k$$

**Figure 2:**
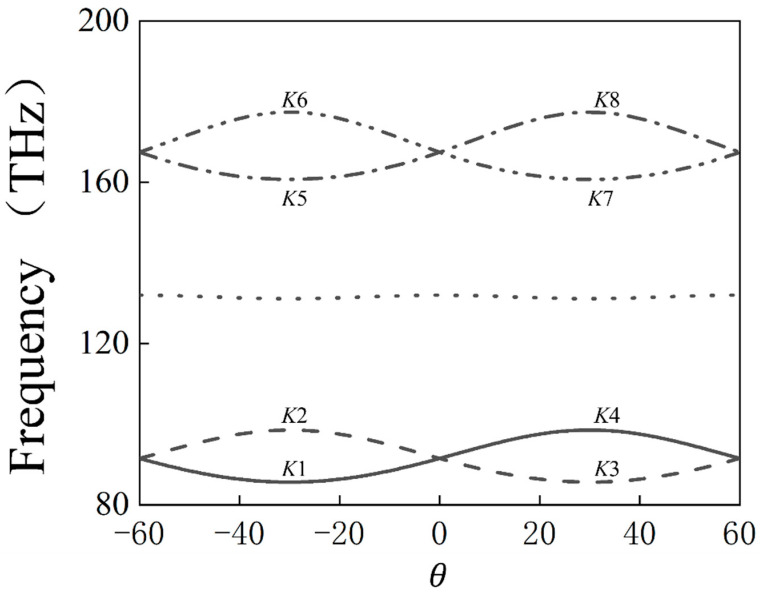
Variation in the band structure at the K-point of a 2D photonic crystal with *C*_3_ symmetry, featuring complete triangular arrangements of triangular photonic units, for *θ* ranging from −60° to 60°.

Figure 2 presents the frequency band gap diagram at the K-point for different rotation angles θ. It is evident that the band gap at the K-point closes and reopens as the angle θ varies, indicating the occurrence of a valley topological phase transition.

**Figure 3:**
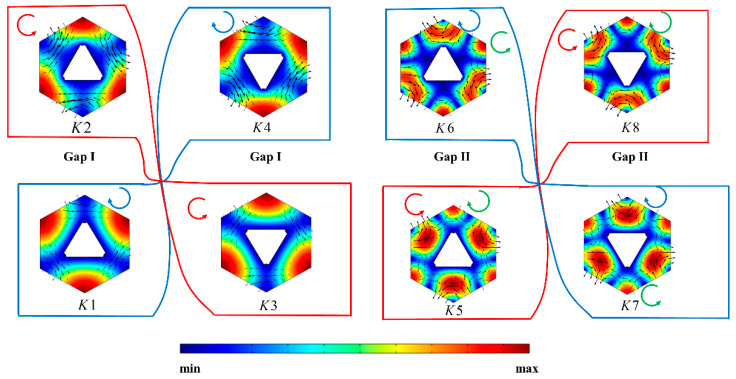
Electric field modes of eigenstate *K*1–*K*8.

The electric field modes of the eigenstates *K*1–*K*8 in Figure 2 are illustrated in Figure 3. The red and blue arrows indicate the direction of energy flow, respectively. Furthermore, a distinct vortex exhibiting chirality, as indicated by green arrows, emerges within Gap II. *K*1, *K*2, *K*5, and *K*6 correspond to the lower and upper bands of Gap I and the lower and upper bands of Gap II at a rotation angle θ of −30° with frequencies of 85.10 THz, 93.39 THz, 160.42 THz, and 173.06 THz, respectively. *K*3, *K*4, *K*7, and *K*8 correspond to the lower and upper bands of Gap I and the lower and upper bands of Gap II at a rotation angle θ of 30° with the same frequencies of 85.10 THz, 93.39 THz, 160.42 THz, and 173.06 THz.

**Figure 4:**
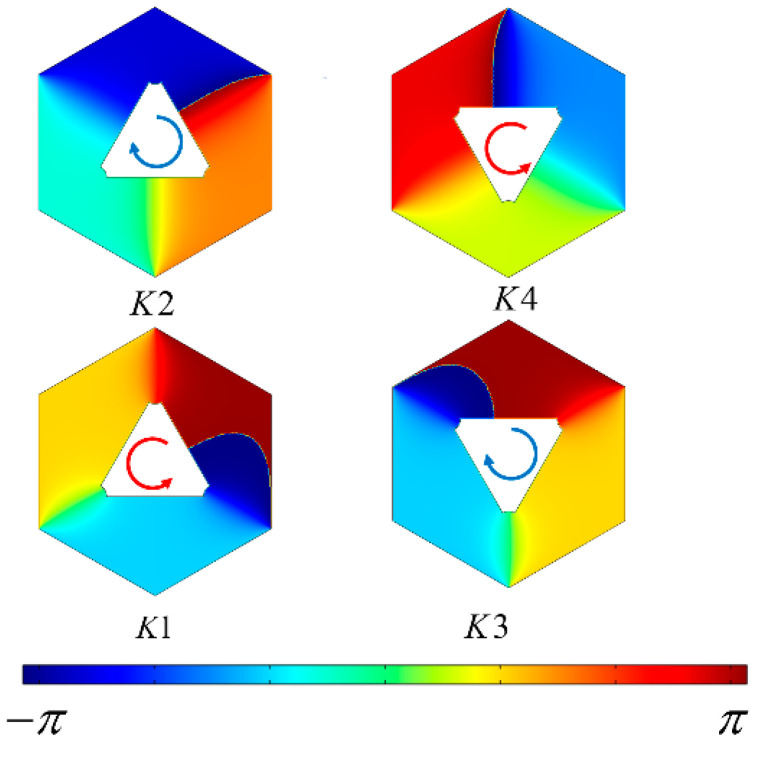
The phase distribution of the electric field *E_Z_* for states *K*1–*K*4 at the K valley is shown. The red arrows and blue arrows indicate the direction of the decreasing phase.

For eigenstates *K*1 and *K*4, in Figure 4, the phase of the electric field *E_Z_* decreases counterclockwise from π to −π. Integration over the K valley yields an orbital angular momentum of l=−1. For eigenstates *K*2 and *K*3, the phase of the electric field decreases clockwise from π to −π, resulting in an orbital angular momentum associated with circular polarization, l=+1.

**Figure 5:**
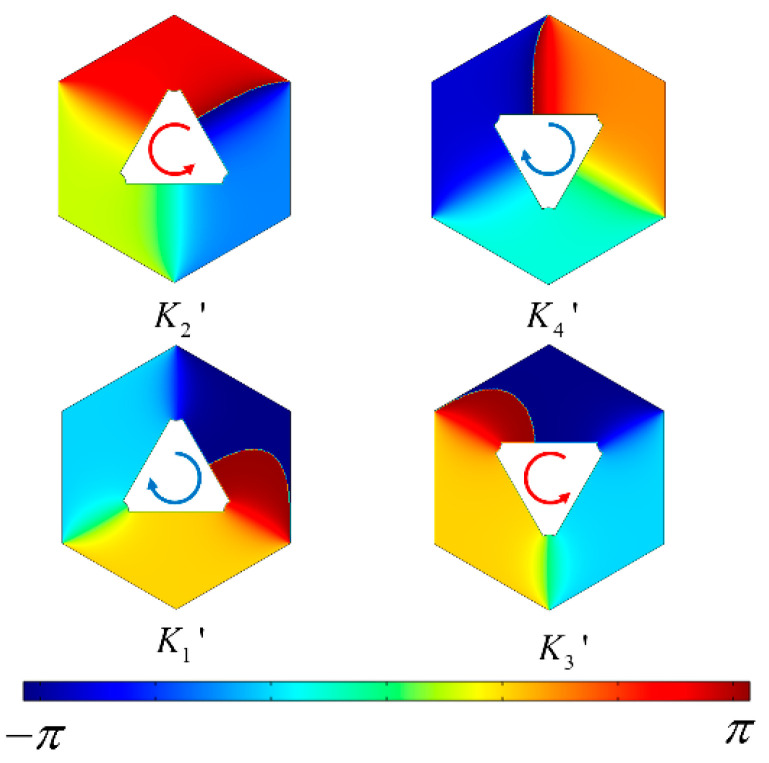
The phase distribution of the electric field *E_Z_* for states *K*1′–*K*4′ at the K′ valley is depicted. The red arrows and blue arrows indicate the direction of decreasing phase.

Figure 5 illustrates the phase distribution of the electric field *E_Z_* for states *K*1′–*K*4′ at the K′ valley. *K*1′ and *K*4′ exhibit right circular polarized phase distributions, while *K*2′ and *K*3′ present left circular polarized phase distributions, opposite to *K*1′–*K*4′.

### 2.2. Excitation of Valley Eigenstates

As demonstrated in Section 2.1, the designed photonic crystal structure exhibits valley topological properties, and the orbital angular momentum carried at the K/K′ points was observed, which is referred to as chirality. Previous studies have explored the utilization of chiral orbital angular momentum for the selective excitation of valley eigenstates. In this study, not only were the valley eigenstates at K/K′ individually excited, but the phase transition of Gap II was leveraged to simultaneously excite the valley eigenstates at both K and K′.

**Figure 6:**
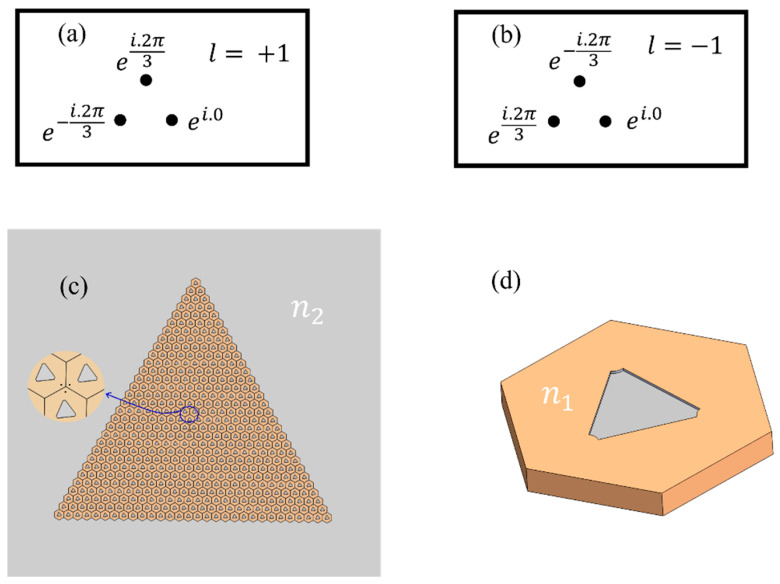
Chiral source carrying orbital angular momentum, composed of three linear current sources. These sources are equidistant and have equal amplitude, with a phase difference of 2π/3 between adjacent sources: (**a**) the phase increases counterclockwise by 2π, carrying a topological charge of +1; (**b**) the phase decreases counterclockwise by 2π, carrying a topological charge of −1; (**c**) schematic diagram of the excitation structure when *θ* is −30°, the enlarged part shows the location of the chiral source; (**d**) schematic of the unit cell structure with $n_{1}=1.6$.

To excite valley eigenstates, a chiral source carrying orbital angular momentum is essential. A chiral source, essential for the excitation of valley eigenstates, is generated by employing three equidistant linear current sources. These sources possess equal amplitudes, but exhibit a phase difference of 2π/3 between adjacent sources. As depicted in Figure 6a, three-point sources increase the phase counterclockwise by 2π, simulating a source with a topological charge of +1 to excite the valley eigenstates. Conversely, the sources in Figure 6b are the opposite of those in Figure 6a, effectively simulating a topological charge of −1. Figure 6c depicts the placement of the chiral source at the center of a triangular structure composed of unit cell structures. Figure 6 illustrates a schematic of the unit cell structure, in which the gray portion represents the metal (PEC) and the yellow portion denotes the dielectric material. At this juncture, $n_{1}=1.6$. The simulation is conducted with the entire structure placed in air, which has a refractive index $n_{2}=1$.

**Figure 7:**
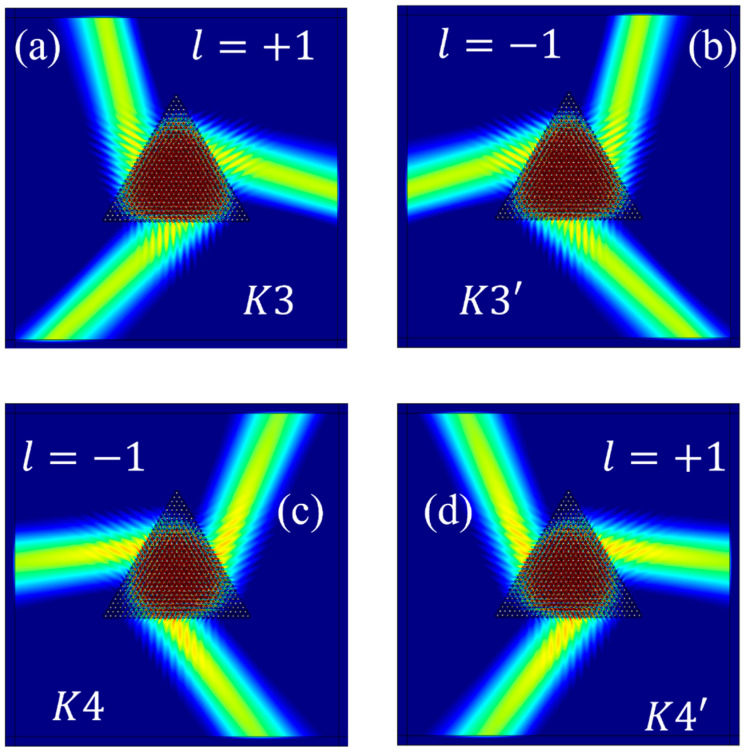
The computational results for the excitation of the valley eigenstates at: (**a**) *K*3, (**b**) *K*3′, (**c**) *K*4, (**d**) *K*4′.

When the rotation angle *θ* of the structure is 30°, the lower band in Gap I, at 85.10 THz, is employed for the excitation of the valley eigenstates. As depicted in Figure 7a, when the source chirality is +1, the eigenstate at the *K*3 valley is excited. Similarly, Figure 7b illustrates that a source chirality of −1 results in the excitation of the eigenstate at the *K*3′ valley. Furthermore, the upper band in Gap I, at 93.39 THz, is also used to excite valley eigenstates. As depicted in Figure 7c, when the source chirality is −1, the eigenstate at the *K*4 valley is excited. Conversely, as illustrated in Figure 7d, when the source chirality is +1, the eigenstate at the *K*4′ valley is excited.

**Figure 8:**
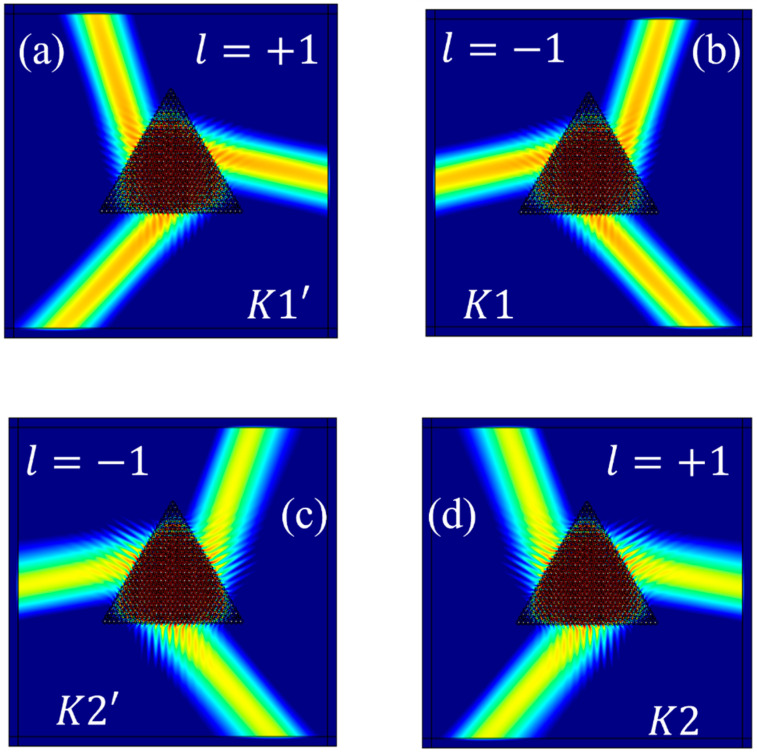
The computational results for the excitation of the valley eigenstates at: (**a**) *K*1′; (**b**) *K*1; (**c**) *K*2′; (**d**) *K*2.

When the rotation angle *θ* of the structure is −30°, the lower band of Gap I, at 85.10 THz, is employed to excite the valley eigenstates. As illustrated in Figure 8a, when the chirality of the source is +1, the eigenstate at the *K*1′ valley is excited. In Figure 8b, when the chirality of the source is −1, the eigenstate at the *K*1 valley is excited. Using the upper band of Gap I, at 93.39 THz, to excite valley eigenstates, Figure 8c illustrates that when the chirality of the source is −1, the eigenstate at the *K*2′ valley is excited. Conversely, in Figure 8d, when the chirality of the source is +1, the eigenstate at the *K*2 valley is excited.

**Figure 9:**
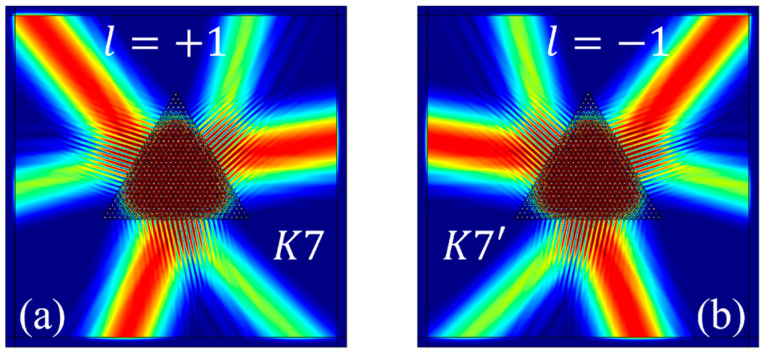
Excitation by the frequency at *K*7 (*K*7′) with a chiral source: (**a**) when a chiral source with +1 is used, the *K*7 state can be excited, which exhibits dual excitation of both the K and K′ valleys. (**b**) When a chiral source with −1 is used, the *K*7′ state is excited, which also demonstrates dual excitation of both the K and K′ valleys.

When the rotation angle *θ* of the structure is 30°, the lower band of Gap II, at 160.42 THz, can simultaneously excite the K valley and the K′ valley. Figure 9a depicts the excited *K*7 valley eigenstate resulting from a source chirality of +1. In this scenario, both the K valley and K′ valley are excited, but the intensity of the excitation in the K valley is stronger than that in the K′ valley, which is consistent with the analysis in Figure 1. Conversely, Figure 9b depicts the excited *K*7′ valley eigenstate resulting from a source chirality of −1. In this case, both the K valley and K′ valley are again excited, but the intensity of the excitation in the K′ valley is stronger than that in the K valley. The *K*8 and *K*8′ valley eigenstates cannot be excited because they are not at extremal points and will be influenced by the eigenfields corresponding to other K values during excitation. Similarly, when the rotation angle *θ* of the structure is −30°, the *K*6 and *K*6′ valley eigenstates cannot be excited either.

**Figure 10:**
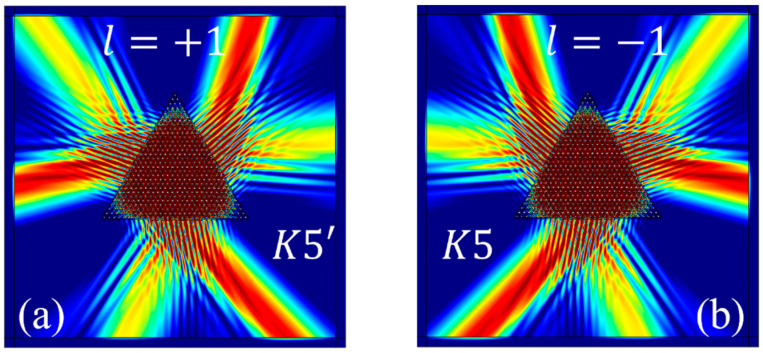
Excitation by the frequency at *K*5 (*K*5′) with a chiral source: (**a**) when a chiral source with +1 is used, the *K*5′ state can be simultaneously excited, which exhibits the dual excitation of both the K and K′ valleys. (**b**) When a chiral source with −1 is employed, the *K*5 state is simultaneously excited, which also demonstrates the dual excitation of both the K and K′ valleys.

When the rotation angle *θ* of the structure is −30°, the lower band of Gap II, at 160.42 THz, enables the simultaneous excitation of the K valley and the K′ valley. When the chirality of the source is +1, the excited *K*5′ valley eigenstate is shown in Figure 10a, where the intensity of the excitation in the K′ valley is stronger than that in the K valley. Conversely, Figure 10b depicts the excited *K*5 valley eigenstate resulting from a source chirality of −1. In this scenario, both the K valley and K′ valley can also be excited, but the intensity of the excitation in the K valley is stronger than that in the K′ valley. This observation aligns with the conclusions derived from the analysis presented in Figure 3.

### 2.3. Momentum-Matching Conditions for the Excitation of Valley Eigenstates

**Figure 11:**
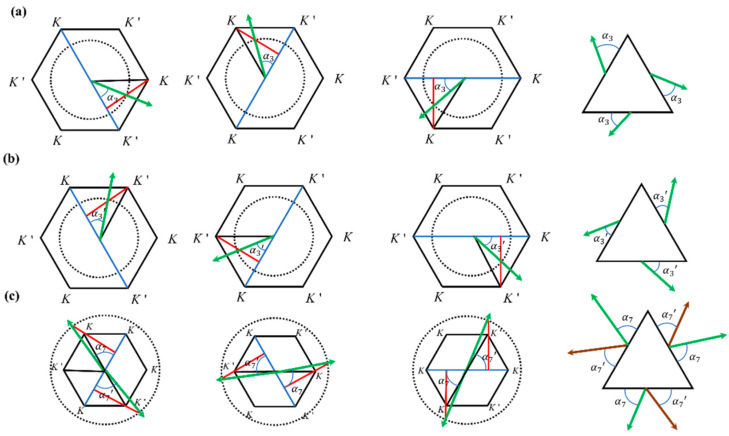
The momentum matching diagram. The black hexagon represents the first Brillouin zone, the dashed black lines indicate the isofrequency contours of the excitation momentum, the red line is the perpendicular line drawn from point K and point K′, and the black triangles denote the boundaries of the structure: (**a**) momentum matching for K valley; (**b**) momentum matching for K′ valley; (**c**) momentum matching for both K and K′ valley.

The excitation of the K valley eigenstate requires satisfying the momentum-matching conditions [24,35]. The K point resides at the vertices of the hexagonal first Brillouin zone; therefore, the momentum at the K valley is given by $\left|K\right|=\frac{2\pi }{a}\times \frac{2}{\sqrt{3}}\times \frac{1}{\sqrt{3}}=\frac{4\pi }{3a}=2.42\times 10^{6}[1/m]$. The excitation momentum is given by $\left|k\right|=\frac{2\pi f}{c/n_{2}}$, where f is the excitation frequency and $n_{2}$ is the refractive index of the environment surrounding the structure. In this case, $n_{2}=1$. Considering the case of θ=30° as an illustrative example, when *K*3 or *K*3′ is excited, $f_{3}=85.10$ THz, resulting in $\left|k_{3}\right|=1.78\times 10^{6}[1/m]$. Figure 11 illustrates the first Brillouin zone, represented by the black hexagon. The black dashed lines indicate the boundaries of the first Brillouin zone, the green and brown lines represent the directions for exciting the valley eigenstates, and the blue line indicates the output boundary. Figure 11a,b depict the isofrequency contours of the excitation momentum, which are enclosed within the first Brillouin zone. The excitation of the *K*3 state is depicted in Figure 11a, and the excitation of the *K*3′ state is shown in Figure 11b. The emission angle is calculated as $\alpha_{3}={\alpha_{3}}^{\prime }=arccos\frac{\left|K\right|}{2\left|k_{3}\right|}=arccos\frac{2.42}{2\times 1.78}=47.18{}^{\circ}$, which matches the numerical simulation results from Figure 7a,b. The analysis for exciting the *K*4 and *K*4′ valley eigenstates is similar to that for *K*3, but the frequency for *K*4 is higher, with $f_{4}=93.39THz$, resulting in $\left|k_{4}\right|=\frac{2\pi f_{4}}{c/n_{2}}=1.96\times 10^{6}[1/m]$. Thus, the isofrequency contour of the exciting momentum is slightly larger than $\left|k_{3}\right|$ but still within the first Brillouin zone defined by $\left|K\right|$. The emission angles are $\alpha_{4}=\alpha_{4^{\prime }}=arccos\frac{\left|K\right|}{2\left|k_{4}\right|}=arccos\frac{2.42}{2\times 1.96}=51.88{}^{\circ}$. Observations show that the emission direction in Figure 7c is rotated clockwise compared to Figure 7b, while in Figure 7d, the emission direction is rotated counterclockwise compared to Figure 7a by a small angle, approximately 51.88°, consistent with the momentum matching results. Similarly, the analysis for *K*1′, *K*1, *K*2′, and *K*2 follows the same method, with conclusions depicted in Figure 8.

In the momentum matching diagram shown in Figure 11c, when *K*7 or *K*7′ is excited, the frequency $f_{7}=160.42THz$ and the corresponding wave vector magnitude is $\left|k_{7}\right|=\frac{2\pi f_{7}}{c/n_{2}}=3.36\times 10^{6}[1/m]$. The isofrequency contour, in this case, encompasses the first Brillouin zone, which results in the simultaneous excitation of the K and K′ valley eigenstates, with the emission angles $\alpha_{7}=\alpha_{7}^{\prime }=arccos\frac{\left|K\right|}{2\left|k_{7}\right|}=arccos\frac{2.42}{2\times 3.36}=68.89{}^{\circ}.$ The green line in the diagram represents the excitation of the K valley, while the brown line indicates the excitation of the K′ valley. These findings are consistent with the numerical simulation results presented in Figure 9a,b. The analysis method for the excitation of the *K*5 and *K*5′ valley eigenstates is similar to that of *K*7 and *K*7′, with the results shown in Figure 10.

## 3. Results and Discussion

### 3.1. Tuning of Excitation Frequency by Refractive Index $n_{1}$

**Figure 12:**
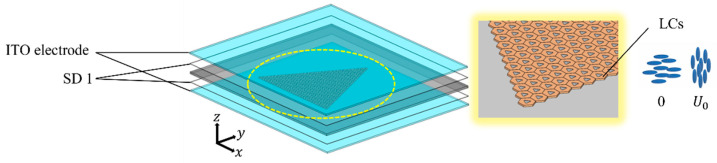
ITO electrodes on dielectric layers. The upper and lower ITO transparent electrodes are spin-coated with SD 1 as orientation layers for liquid crystals. When the voltage is 0, the orientation of the LC is in the horizontal state. When a bias voltage of *U*_0_ is applied, the orientation of LC changes from horizontal to vertical.

For the tunable valley eigenstate structure using liquid crystals, as shown in Figure 1, the surface of the dielectric layer is equipped with ITO electrodes, and the upper and lower ITO electrodes are spin-coated with SD 1 as the orientation layer for liquid crystals, as illustrated in Figure 12. Through the variation in the electric field, the refractive index $n_{1}$ can be modulated within the range of 1.5 to 1.8 in the THz frequency regime [31]. The photonic band structures for rotation angles θ=30° and θ=−30° are identical. Given that the third and fifth bands do not support excitations of valley eigenstates, we focus on the edges of the two bands of Gap I and the lower band of Gap II.

**Figure 13:**
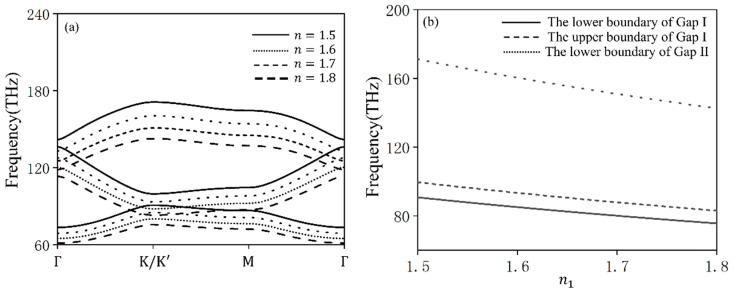
Tunable frequencies of the valley eigenstate structure: (**a**) band curves corresponding to *n*_1_ values of 1.5, 1.6, 1.7, and 1.8, respectively; (**b**) variation in the energy band at K (K′) with changes in $n_{1}$.

When $n_{1}$ is set to 1.5, 1.6, 1.7, and 1.8, the corresponding bands are shown in Figure 13a, and the variation in the bands at the K(K′) point with $n_{1}$ is illustrated in Figure 13b. As demonstrated in the figures, modulating the refractive index via applied voltage enables the tunable frequencies of the valley eigenstate structure to transition from three discrete frequency points (corresponding to *K*1 (*K*3), *K*2 (*K*4), and *K*5 (*K*7) in Section 2) to three broadbands: 75.64 THz–90.77 THz, 83.01 THz–99.61 THz, and 142.60 THz–171.12 THz. This transition significantly broadens the operational frequency range of the valley eigenstate structure. The range from 75.64 THz to 99.61 THz can be used to control the excitation of either the K valley or the K′ valley independently by adjusting the rotation angle θ and the chirality of the excitation source. In contrast, at excitation frequencies from 142.60 THz to 171.12 THz, both the K valley and the K′ valley can be excited simultaneously.

**Figure 14:**
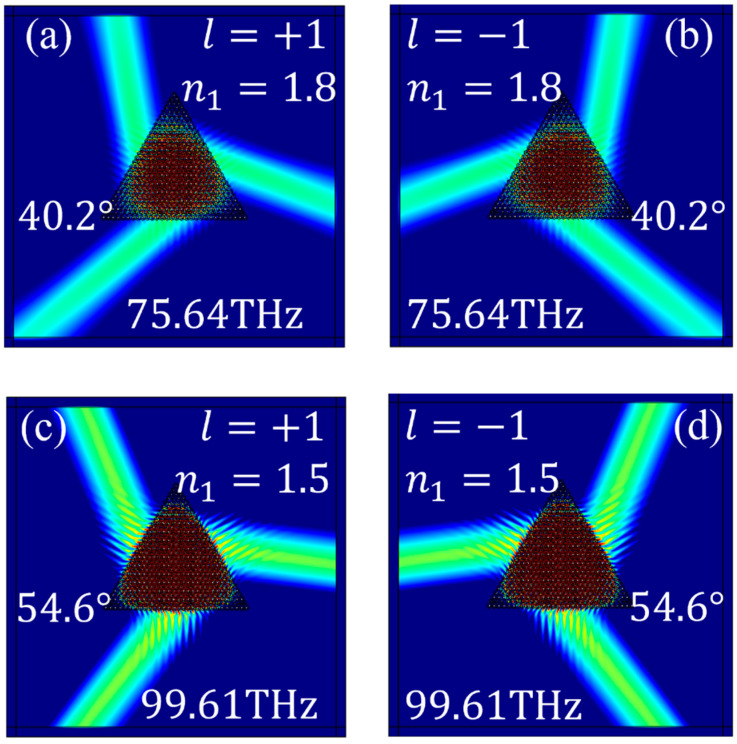
Excitation diagrams of Gap I under different $n_{1}$: (**a**) eigenstate at a frequency of 75.64 THz when $n_{1}\;$= 1.8 and l=+1; (**b**) eigenstate at a frequency of 75.64 THz when $n_{1}\;$= 1.8 and l=−1; (**c**) eigenstate at a frequency of 99.61 THz when $n_{1}\;$= 1.5 and l=+1; (**d**) eigenstate at a frequency of 99.61 THz when $n_{1}\;$= 1.5 and l=−1.

To illustrate this behavior, we consider the case of *θ* = 30° and examine the excitation diagrams presented in Figure 14 under various conditions: Figure 14a depicts the excitation of the K point valley eigenstate at a frequency of 75.64 THz when $n_{1}\;$= 1.8 and the chirality of the source is +1; Figure 14b depicts the excitation of the K′ point valley eigenstate at a frequency of 75.64 THz when $n_{1}$=1.8 and the chirality of the source is −1; Figure 14c depicts the excitation of the K point valley eigenstate at a frequency of 99.61 THz when $n_{1}\;$= 1.5 and the chirality of the source is +1; Figure 14d illustrates the excitation of the K′ point valley eigenstate at a frequency of 99.61 THz when $n_{1}\;$= 1.5 and the chirality of the source is −1. Based on the momentum-matching conditions discussed in Section 2.3, the calculated emission angles are 40.2° at a frequency of 75.64 THz and 54.6° at a frequency of 99.61 THz. These calculated emission angles are in agreement with the simulation results presented in Figure 14.

**Figure 15:**
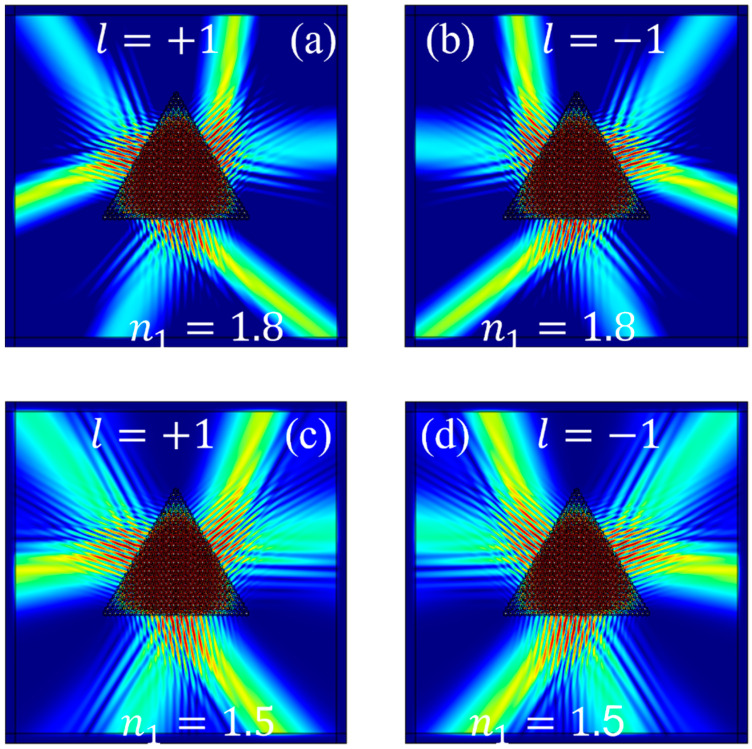
Excitation diagrams of Gap II under different $n_{1}$: (**a**) eigenstate at a frequency of 142.60 THz when $n_{1}\;$= 1.8 and l=+1; (**b**) eigenstate at a frequency of 142.60 THz when $n_{1}\;$= 1.8 and l=−1; (**c**) eigenstate at a frequency of 171.12 THz when $n_{1}\;$= 1.5 and l=+1; (**d**) eigenstate at a frequency of 171.12 THz when $n_{1}\;$= 1.5 and l=−1.

Figure 15a depicts the excitation schematic of the valley states at the K and K′ points at a frequency of 142.60 THz when $n_{1}$ = 1.8 and the chiral source of +1; Figure 15b depicts the excitation schematic of the valley states at the K and K′ points at 142.60 THz when $n_{1}$ = 1.8 and the chiral source is -1. Figure 15c depicts the excitation schematic of the valley states at the K and K′ points at a frequency of 171.12 THz when $n_{1}$ = 1.5 and the chiral source is 1; Figure 15d depicts the excitation schematic of the valley states at the K and K′ points at 171.12 THz when $n_{1}$ = 1.5 and the chiral source is −1. It can be observed that as the frequency increases, the emission angle $\alpha_{7}$ becomes larger and the two beams of outgoing light on the same side come closer to each other. The diffraction effect of the two beams with the same frequency also becomes more pronounced, which is consistent with the momentum-matching conditions described in Section 2.3.

### 3.2. Altering the Emission Angle and Switching Modes by Refractive Index $n_{2}$

As depicted in Figure 6c, the material with a refractive index of $n_{2}$ is situated within an environment that encompasses the entire structure. By placing the structure in environments with varying refractive indices, it is possible to alter the refractive index $n_{2}$.

In this case, taking $n_{1}\;$= 1.6 as an example, we know from Section 2.3 that the emission angle is given by $arccos\frac{K}{2\left|k\right|}=arccos\frac{c}{3a\cdot f\cdot n_{2}}$. Our calculations reveal that the emission angle increases monotonically with increasing frequency. Continuing with the example of the *K*₃ valley state at θ=30°, Section 2.3 also indicates that when $\left|k\right|>\left|K\right|$, the valley states at K and K′ points are simultaneously excited, as shown in Figure 7a,b. Conversely, when $\left|k\right|<\left|K\right|$, if the chirality of the source is +1, the K point valley state is excited; if the chirality is −1, the K′ point valley state is excited. By calculating $\left|K\right|=\frac{4\pi }{3a}=\left|k\right|=\frac{2\pi f}{c/n_{2}}$, we find that when $f_{3}=85.10$ THz, the critical refractive index $n_{2}$ is approximately 1.35.

**Figure 16:**
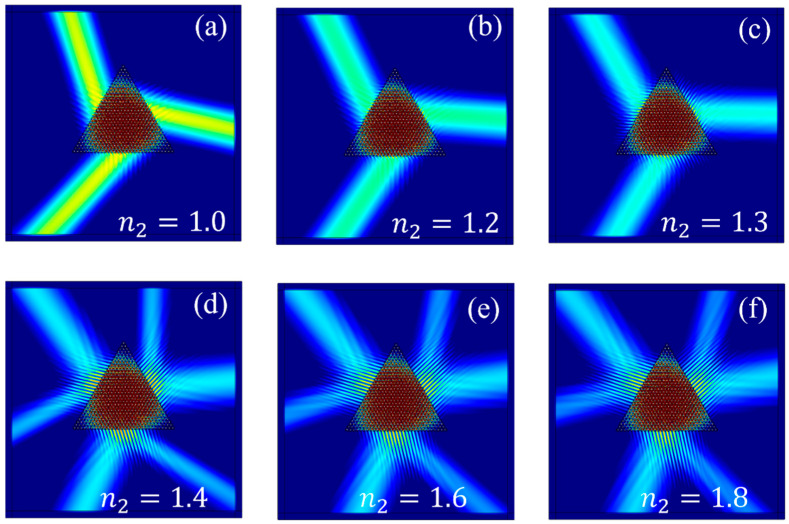
Excitation diagrams of Gap II under different $n_{2}$ with $n_{1}=1.6$, $f_{3}=85.10$ THz, and l=+1: (**a**) eigenstate at $n_{2}$= 1.0; (**b**) eigenstate at $n_{2}$=1.2; (**c**) eigenstate at $n_{2}\;$= 1.3; (**d**) eigenstate at $n_{2}\;$= 1.4; (**e**) eigenstate at $n_{2}\;$= 1.6; (**f**) eigenstate at $n_{2}\;$= 1.8.

Figure 16a–f depict the valley state excitations for six representative refractive index values: $n_{2}$ = 1.0, 1.2, 1.3, 1.4, 1.6, and 1.8. These results demonstrate that, at a fixed frequency, increasing the applied voltage to gradually raise the refractive index $n_{2}$ results in a corresponding increase in the emission angle α. When $n_{2}$ exceeds a critical value, another valley state can be simultaneously excited, which is consistent with the conclusions drawn in Section 2.3. This structure holds the potential for facilitating the design of optical logic gates and tunable filters, with significant implications for applications in photonic computing and optical cloaking.

## 4. Conclusions

This paper presents a topological photonic crystal structure with a triangular lattice that enables selective excitation of the K and K′ valleys at frequencies of 85.10 THz and 93.39 THz, respectively, by utilizing incident light with different helicities. Furthermore, simultaneous excitation of both the K and K′ valleys is achievable at a frequency of 160.42 THz. The simulated emission angles are in agreement with the theoretical calculations based on momentum-matching conditions.

By integrating liquid crystal materials into the structural regions of the topological photonic crystal, the portion of the structure that controls the refractive index $n_{1}$ can extend the excitation frequency points of 85.10 THz, 93.39 THz, and 160.42 THz into two broad spectral bands of 75.64 THz–99.61 THz and 142.60 THz–171.12 THz. The broadband from 75.64 THz to 99.61 THz can be used to separately excite the K and K′ valleys using vortex light sources of different helicities, while the frequency range of 142.60 THz to 171.12 THz allows for the simultaneous excitation of both K and K′ valleys, significantly broadening the applicable frequency range of this structure.

Furthermore, the portion that controls the refractive index $n_{2}$ enables the adjustment of the emission angle for a single fixed frequency and allows for the independent excitation of either the K valley or K′ valley, as well as their simultaneous excitation. This enhanced control over valley state excitation holds significant potential for applications in areas such as photonic computing and tunable filtering.

## Data Availability

The original contributions presented in the study are included in the article; further inquiries can be directed to the corresponding author.
